# Core pluripotency factors promote glycolysis of human embryonic stem cells by activating GLUT1 enhancer

**DOI:** 10.1007/s13238-019-0637-9

**Published:** 2019-05-31

**Authors:** Lili Yu, Kai-yuan Ji, Jian Zhang, Yanxia Xu, Yue Ying, Taoyi Mai, Shuxiang Xu, Qian-bing Zhang, Kai-tai Yao, Yang Xu

**Affiliations:** 1 Cancer Research Institute, Guangdong Provincial Key Laboratory of Cancer Immunotherapy, School of Basic Medical Sciences, Southern Medical University, Guangzhou, 510515 China; 2 The Eighth Affiliated Hospital, Sun Yat-sen University, Shenzhen, 518033 Guangdong China

**Keywords:** human embryonic stem cell, pluripotency factors, metabolism, Glut1, enhancer, promoter, epigenetics, chromosome interaction

## Abstract

Human embryonic stem cells (hESCs) depend on glycolysis for energy and substrates for biosynthesis. To understand the mechanisms governing the metabolism of hESCs, we investigated the transcriptional regulation of glucose transporter 1 (GLUT1, SLC2A1), a key glycolytic gene to maintain pluripotency. By combining the genome-wide data of binding sites of the core pluripotency factors (SOX2, OCT4, NANOG, denoted SON), chromosomal interaction and histone modification in hESCs, we identified a potential enhancer of the GLUT1 gene in hESCs, denoted GLUT1 enhancer (GE) element. GE interacts with the promoter of GLUT1, and the deletion of GE significantly reduces the expression of GLUT1, glucose uptake and glycolysis of hESCs, confirming that GE is an enhancer of GLUT1 in hESCs. In addition, the mutation of SON binding motifs within GE reduced the expression of GLUT1 as well as the interaction between GE and GLUT1 promoter, indicating that the binding of SON to GE is important for its activity. Therefore, SON promotes glucose uptake and glycolysis in hESCs by inducing GLUT1 expression through directly activating the enhancer of GLUT1.

**Electronic supplementary material:**

The online version of this article (10.1007/s13238-019-0637-9) contains supplementary material, which is available to authorized users.

## INTRODUCTION

Human embryonic stem cells (hESCs) can undergo unlimited self-renewal and maintain the pluripotency to differentiate into all lineages of cells of the human body (De Los Angeles et al., 2015). This metabolic signature of pluripotency is similar to the Warburg effect in human cancers and is primarily dependent on glycolysis (Shyh-Chang and Daley, 2015). In this context, glycolysis produces ATP at a faster rate than oxidative phosphorylation, and glycolytic intermediates are biosynthesis substrates required for unlimited self-renewal of hESCs (Shyh-Chang and Daley, 2015). In addition, glycolysis produces acetyl-CoA to promote histone acetylation, which is required to maintain the epigenetics of hESCs (Moussaieff et al., 2015). The transition from oxidative phosphorylation to glycolysis also promotes the reprogramming of induced pluripotent stem cells (iPSCs) (Folmes et al., 2011). While the core transcriptional factors SRY (sex determining region Y)-box 2 (SOX2), octamer-binding transcription factor 4 (OCT4), and NANOG, collectively denoted SON, are required to maintain pluripotency (Chen et al., 2008), their roles in maintaining the metabolic profile of ESCs remain unclear.

The increase of glucose uptake is required to maintain high levels of glycolysis. GLUT1 plays a key role in glucose uptake in many cell types including ESCs and cancer cells (Shyh-Chang and Daley, 2015; Ancey et al., 2018). The expression of GLUT1 is significantly increased during early embryonic development from the two-cell stage to the blastocyst stage (Morita et al., 1994). Consistent with this finding, GLUT1 is also highly expressed in pluripotent stem cells (Shyh-Chang and Daley, 2015). Studies of GLUT1-deficient and GLUT1-haplodeficient mouse ESCs indicated that GLUT1 is required for the survival of pluripotent stem cells by maintaining high levels of glycolysis (Ohtsuki et al., 2006).

Enhancers are clusters of distal DNA sequences that can increase transcription of their target gene(s) in cis in eukaryote (Pennacchio et al., 2013). The activity of enhancers in the human genome is time- and cell type-dependent. Epigenetic markers commonly used to identify active enhancers include histone H3 acetylated at lysine 27 (H3K27ac) and H3 monomethylated at K4 (H3K4me1) (Deng et al., 2012; Calo and Wysocka, 2013). Chromatin Interaction Analysis with Paired-End-Tag sequencing (ChIA-PET) and Hi-C (Genome-wide 3C) demonstrate that enhancer–promoter interaction through chromosomal looping is necessary for transcriptional activation of genes (Dekker et al., 2002; Dostie et al., 2006; Zhao et al., 2006; Fullwood et al., 2009; Lieberman-Aiden et al., 2009). High-resolution interaction data of ChIA-PET can provide the information of chromatin interaction (Barutcu et al., 2016). When combining with the chromatin immunoprecipitation sequencing (ChIP-seq) data of enhancer histone markers, cohesin ChIA-PET data can help to accurately identify the enhancer-promoter loops (Ji et al., 2016).

In this study, we identified a novel enhancer for GLUT1 in hESCs, which appeared to be evolutionarily conserved in other pluripotent stem cells and cancer cells. In addition, we demonstrate that the binding site of SON within this enhancer is important for the enhancer activity and glucose uptake. Therefore, SON plays important roles in maintaining pluripotency by inducing glycolysis in hESCs.

## RESULTS

### To identify the enhancer of the GLUT1 gene in hESCs

**Figure 1:**
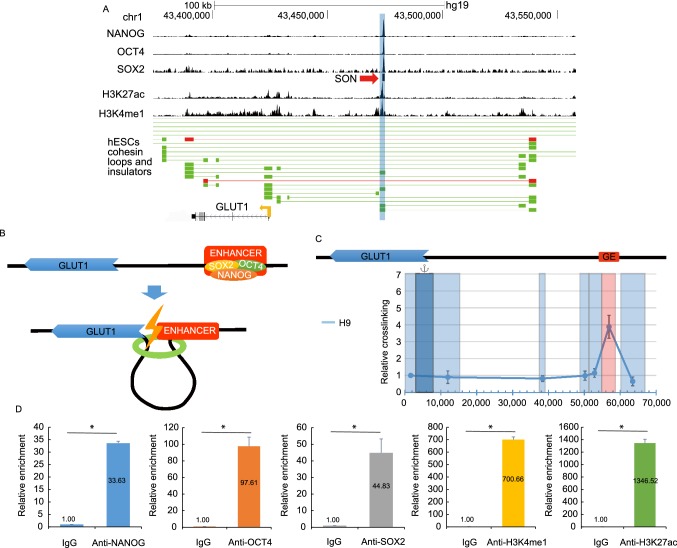
**Identification of the*GLUT1*enhancer, denoted GE, in hESCs**. (A) Integrated analysis to predict the enhancer of GLUT1 in H9 hESCs. Based on the epigenetic signature of enhancers, the blue shaded region is the predicted enhancer of GLUT1 in hESCs. Insulator loop (red line) and cohesin loop (green line) involving the enhancer and promoter of GLUT1 are displayed, the blocks connected by a horizontal line show the interaction of two regions of the genome. Based on the ChIP-seq data, the binding sites for NANOG, OCT4, SOX2 as well as H3K27ac and H3K4me1 around GE are displayed at the top. The co-binding site of SOX2, OCT4 and NANOG (SON) is indicated with a red arrowhead. (B) A hypothetical model how the promoter and enhancer of the *GLUT1* gene interact in hESCs. (C) 3C analysis confirmed the long-distance interaction between the promoter and enhancer of the *GLUT1* gene. The GLUT1 promoter is indicated by dark blue and target restriction fragments light blue. GE-containing restriction fragment is shaded red. Primers were all forward orientation and positioned at the right end of each restriction fragment. Relative cross-linking value for each restriction fragment was plotted over the 70 kb genomic DNA fragment. Data represent mean ± SEM. *n* = 3. The value of the crosslinking between promoter and the nearest neighboring fragment is arbitrarily set to 1. (D) ChIP-qPCR assay was used to confirm the binding of SON to GE and the epigenetic signature of GE in hESCs. Data represent mean + SEM. *n* = 3

To identify the potential enhancer of the GLUT1 gene in hESCs, we analyzed the ChIA-Pet data of SMC1 and the genome-wide epigenetic histone markers characteristics of enhancer in hESCs. We identified some genomic regions that could interact with the promoter of GLUT1 and were marked with both H3K27ac and H3K4me1 (Fig. 1A). The one with the highest intensity of the epigenetic histone markers was located about 50 kb downstream of the GLUT1 promoter (Fig. 1A and 1B). We predicted that this genomic region was the enhancer for the GLUT1 gene in hESCs, denoted GE. The long-range interaction between the promoter of GLUT1 and GE was confirmed with 3C assay (Figs. 1C, and S1A). Another reason we focused on GE was that the analysis of the ChIP-seq data of SOX2, OCT4, and NANOG denoted SON, in hESCs indicated the co-binding of SON to GE (Fig. 1A and 1B). The histone epigenetic markers of GE and the binding of SON of GE were confirmed with ChIP-qPCR (Fig. 1D).

### GE is important for the expression of GLUT1 in hESCs

**Figure 2:**
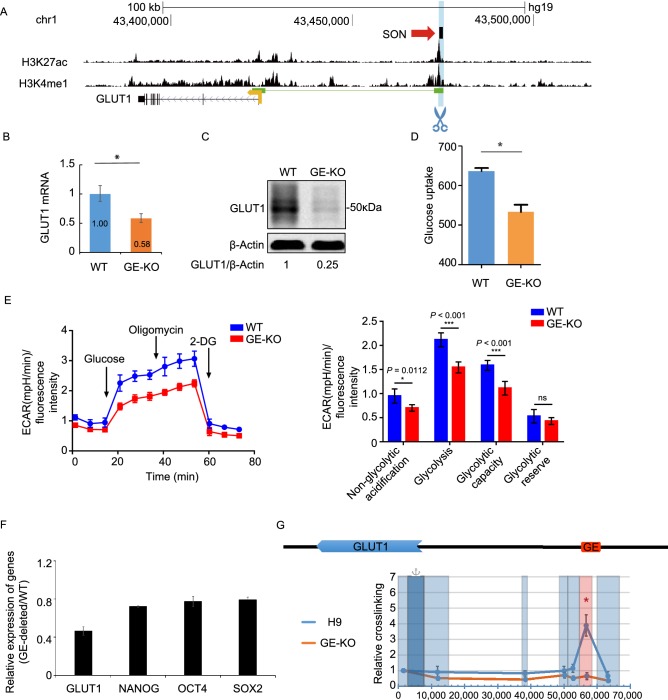
**The predicted enhancer of the*GLUT1*gene (GE) is required for the expression of GLUT1 in hESCs**. (A) The strategy to delete GE in H9 hESCs using CRISPR/CAS9 technology. The top panel shows the SON binding site and the epigenetic profiles for H3K27ac and H3K4me1. The deleted region is indicated in light blue. (B) Deletion of GE in hESCs reduced the mRNA levels of GLUT1. GE-deleted hESCs are denoted GE-KO hESCs. Data represent mean ± SEM. *n* = 3. (C) Deletion of GE in hESCs reduced the protein levels of GLUT1. (D) Deletion of GE in hESCs reduced glucose uptake. Data represent mean ± SD. *n* = 4. (E) Deletion of GE in hESCs significantly reduced their ECAR. Data represent mean ± SD. *n* = 6. (F) The deletion of GE reduced the mRNA expression levels of pluripotency genes in hESCs. Data represent mean + SD. *n* = 3. (G) Deletion of GE greatly reduced the long-distance chromosomal interaction between promoter and GE region of the *GLUT1* gene. The GLUT1 promoter area is indicated by dark blue and the target restriction fragments light blue. GE-containing restriction fragment is indicated by red color. Primers were all forward orientation and positioned at the right end of each restriction fragment. Data represent mean ± SEM. *P ≤ 0.05 by two-tailed Student’s *t* test comparing WT to GE-KO. *n* = 3

To determine whether GE could function as an enhancer of GLUT1, we deleted GE from the genome using CRISPR/CAS9 technology with two sgRNAs flanking the GE element as previously described (Rong et al., 2014). The analysis of the homozygous GE-KO (GE knockout) hESCs indicated that the deletion of GE decreased the expression of the GLUT1 mRNA and protein in hESCs (Figs. 2A–C, S1B-E). Consistent with this finding, when compared to those of WT hESCs, the glucose uptake and glycolysis of GE-KO hESCs were significantly reduced (Fig. 2D and 2E). The mRNA expression levels of the key pluripotency factors NANOG, OCT4, SOX2 in GE-deleted hESCs were lower than those in WT hESCs, indicating that GE-deletion impaired the pluripotency of hESCs (Fig. 2F). GE-deletion abolished the long-range interaction between the GE-containing region and GLUT1 promoter, further confirming that GE is the enhancer of GLUT1 gene (Fig. 2G). In summary, these data demonstrate that GE functions as an enhancer of GLUT1 gene in hESCs and is required to maintain the glycolysis and pluripotency of hESCs.

### The SON binding site within GE is important for the enhancer function of GE

**Figure 3:**
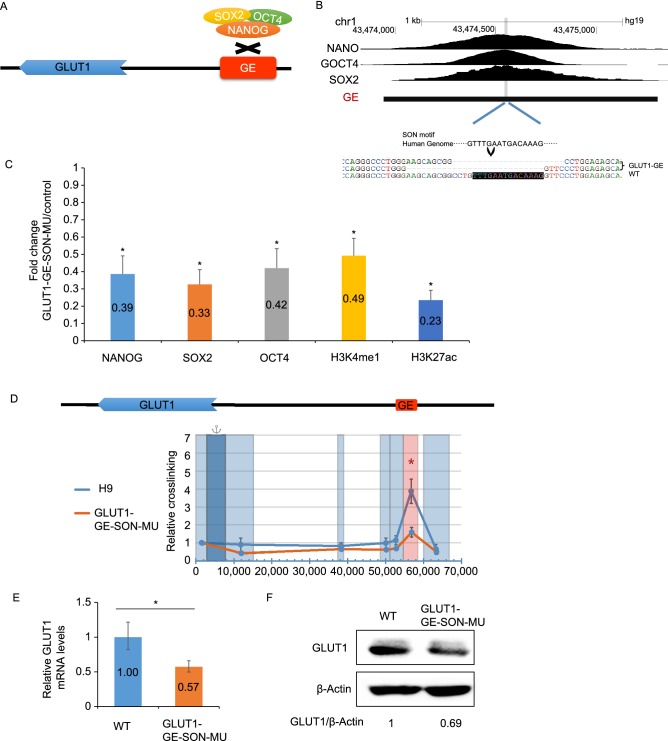
**SON-binding motif within GE is required for GE activity in activating*GLUT1*expression**. (A) Schematic strategy to disrupt the SON binding site within GE. (B) Identification of the core binding sequence of the SON binding site based on the ChIP-seq data of SON in hESCs and disruption of the SON binding motif in hESCs. The sequences of GLUT1-GE-SON-MU hESCs are shown below, and the binding motif of SON is shaded black. (C) The disruption of the SON binding motif within GE in hESCs reduced the binding of SON to GE and the enhancer-specific epigenetic signature of GE as confirmed by ChIP-qPCR assay. Data represent mean + SEM. *n* = 3. (D) The disruption of the SON binding motif reduced the long-range interaction between the promoter and GE of GLUT1. The GLUT1 promoter is indicated by dark blue and target restriction fragments light blue. GE-containing fragment is indicated by red box. Data represent mean ± SEM. *n* = 3. *P < 0.05. (E) The disruption of the SON site reduced the mRNA levels of the GLUT1 gene. Data represent mean ± SEM. *n* = 3. (F) The disruption of the SON site reduced the protein levels of GLUT1 in hESCs

While the knockdown of NANOG, OCT4 and SOX2 individually fails to reduce the expression of GLUT1 before the onset of differentiation (Wang et al., 2012), it remains possible that SON plays important but redundant roles in activating GE. The binding site of SON within GE, TTTGAATGACAAAG, was predicted based on the motif of OCT4-SOX2-TCF-NANOG in HOMER database and the peak of ChIP-seq data of SON in hESCs (Fig. 3B). To investigate the roles of SON binding site within GE, we targeted mutated the SON binding site within in the genome of hESCs (Fig. 3B). The homozygous mutation of SON reduced the binding of SON to GE and the levels of the epigenetic markers of the enhancer (Fig. 3C). In addition, the deletion of SON binding site within GE decreased the expression of GLUT1 (Fig. 3E and 3F). Therefore, SON is important to induce the expression of GLUT1 through direct activation of GE.

### GE-deleted hESCs are defective in GLUT1 expression during differentiation

**Figure 4:**
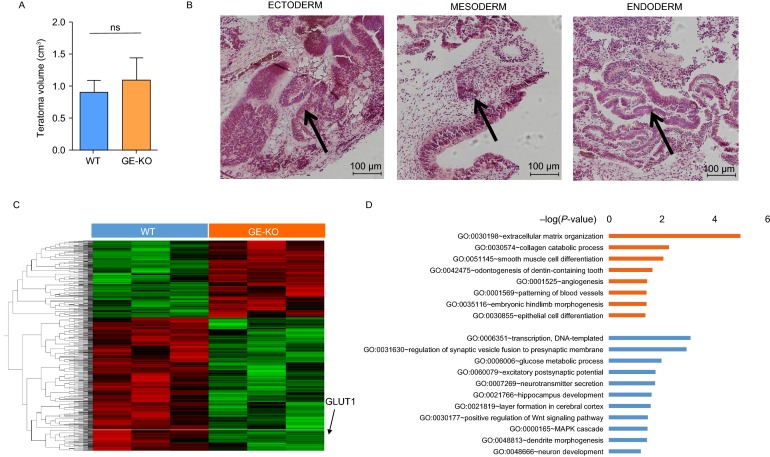
**The deletion of GE reduced the expression of*GLUT1*and genes of neural lineages in teratomas formed by hESCs**. (A) The volume of teratomas formated by GE-KO and wild type control hESCs. The formula V = ab^2^/2 was used to calculate the tumor or teratoma volume (V). The length (a) and width (b) of the teratoma were measured with calipers. (B) Cells derived from each of the three germ layers were present in the teratomas formed by GE-KO hESCs in NSG mice. (C) The heat maps showing the differentially expressed genes in the teratomas formed by control parental hESCs and GE-KO hESCs. *n* = 3. (D) Enriched GO biological process of up-regulated genes (orange) and down-regulated genes (blue) in teratomas formed by GE-KO hESCs. The X-axis of histogram is −log10(*P* value) of individual terms calculated by right-sided hypergeometric test and corrected with Bonferroni. GO categories are indicated on Y-axis

To evaluate the physiological roles of GE in the differentiation of hESCs, we used RNA-seq to compare the global gene expression in the teratomas formed by hESCs and GE-deleted hESCs. There was no difference in the size of the teratomas formed by hESCs and GE-KO hESCs (Fig. 4A). Teratomas formed by hESCs and GE-KO hESCs contained the cells derived from each of the three germ layers, indicating that the deletion of the GLUT1 enhancer did not abolish the pluripotency of hESCs (Fig. 4B). We identified 982 differentially expression genes (DEG, *P*-value < 0.05) between the RNA samples of teratomas formed by hESCs and GE-KO hESCs (Fig. 4C). The expression of GLUT1 in the teratomas formed by GE-KO hESCs was decreased to about 40% of that of WT hESCs. Gene ontology (GO) biological process enrichment analysis of DEGs indicated that the global expression of genes involved in neural differentiation and glucose metabolic process were reduced in teratomas formed by GE-KO hESCs when compared to that in teratomas formed by WT hESCs (Fig. 4D). These findings suggest that GE activates the expression of GLUT1 during the differentiation of hESCs and neural development.

### GE is conserved in iPSCs and human cancer cells

**Figure 5:**
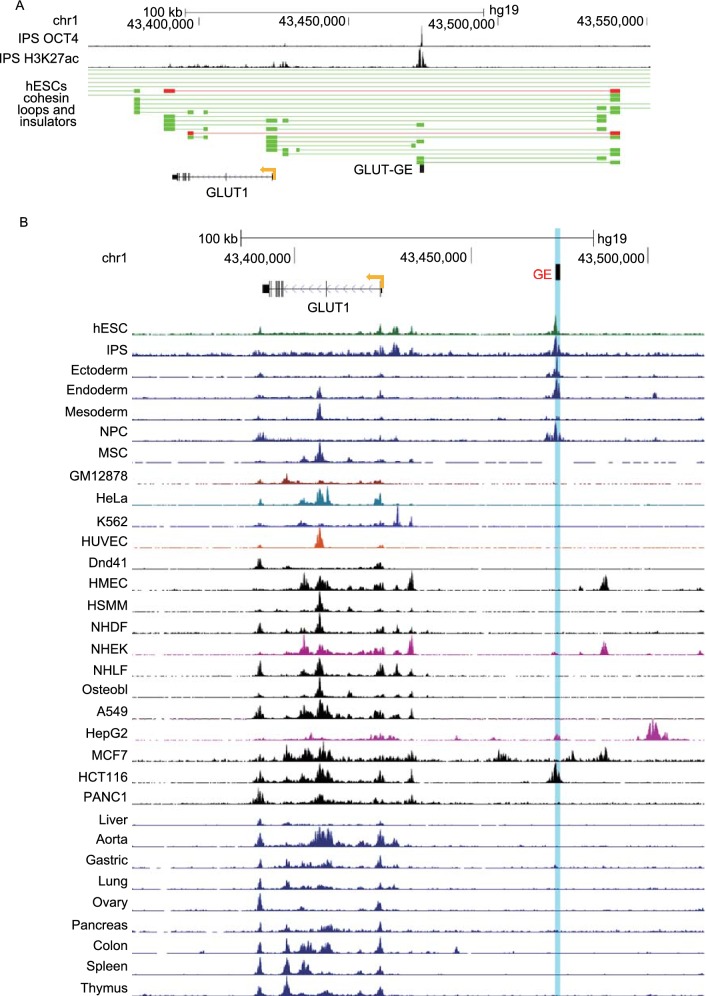

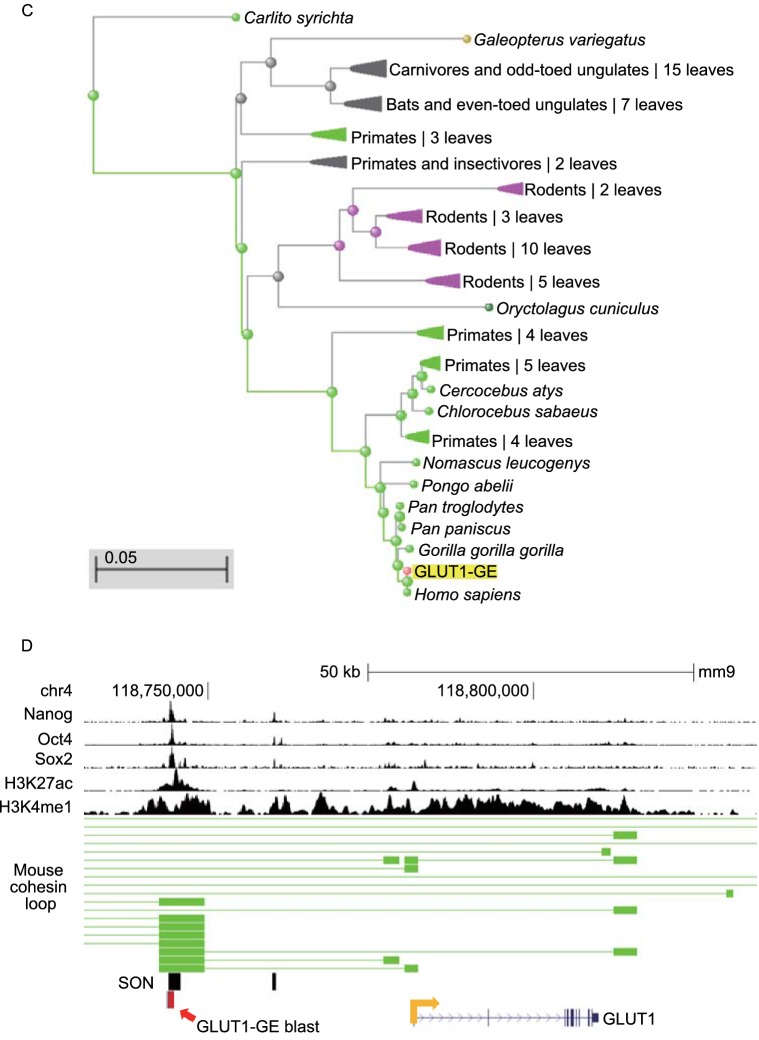
**The conservation of GE in various human cells and higher mammals**. (A) Integrated analysis of the activity of GE in human IPSC lines. Insulators (red lines) and interactions (green lines) of human iPSCs involving the enhancer and promoter of *GLUT1* are displayed at bottom. Binding profiles for OCT4 and H3K27ac of IPSCs are displayed at the top. (B) GE is active in human iPSCs, ectoderm, endoderm, neural progenitor cells (NPCs), mesenchymal stem cells (MSCs), and certain human cancer cells as indicated by the epigenetic marker (H3K27ac) labeled in blue. (C) Fast minimum evolution tree was used to reveal the evolutionary relationship of GE. (D) GE is active in mouse ESCs. The epigenetic signature (H3K27ac and H3K4me1) of GE (blue box) and the binding profiles of SON are conserved in mouse ESCs. The predicted interaction sites between the enhancer and promoter of the Glut1 gene in mouse ESCs are indicated by green boxes. The conserved region of the mouse and human GE is indicated with a red arrowhead

Considering that pluripotent stem cells (hESCs and iPSCs) and human cancer cells share their dependence on glycolysis for survival, we also analyzed the enhancer-specific epigenetic signature of GE in iPSCs, different germ layer derived from hESCs, human cancer cell lines and tissues, indicating that GE is also active in IPSCs, ectoderm, endoderm, NPC (neural progenitor cells), and human cancer cell lines (HepG2 and HCT116) (Fig. 5A and 5B). The finding that GE is active in ectoderm and neural progenitor cells further supports the notion that GE is involved in neural differentiation. In addition, the enhancer-specific epigenetic signature of GE is also conserved in other species such as primates, rodents, carnivores, odd-toed ungrlates and even-toed ungulates (Fig. 5C). As the representative of rodents, the genomic structure of the mouse Glut1 and GE was similar to that of human (Fig. 5D). Therefore, GE is an evolutionarily conserved enhancer to control the expression of GLUT1.

## DISCUSSION

GLUT1 is important for high levels of glucose uptake to maintain glycolysis of ESCs and human cancer cells (Ancey et al., 2018). Therefore, it is important to reveal the mechanisms how hESCs regulate the expression of GLUT1. We identified an enhancer element of GLUT1 (GE) in hESCs that is required for the optimal levels of GLUT1 expression. To understand how this enhancer is activated in hESCs, we used the genome-wide ChIP-seq data to identify the proteins that can bind to GE and found that the pluripotency factors Sox2, Oct4 and Nanog all bind to GE. By disrupting this binding site of SON in the genome of hESCs, we showed that the binding sites are important for the binding of SON to GE, the long-range interaction between enhancer and promoter, and the activity of GE. Therefore, SON is important for inducing the expression of GLUT1 by activating its enhancer.

Mutations in GLUT1 can cause GLUT1 deficiency syndrome, which led a neurologic disorder and epilepsy in human (Schneider et al., 2009; Striano et al., 2012). In mouse model, homozygous loss of GLUT1 is associated with embryonic lethality and heterozygous mouse performed incoordination, hypoglycorrhachia and microencephaly, such as epilepsy (Wang et al., 2006; Zheng et al., 2010). In our result, decrease of GLUT1 in GE-deleted teratomas cause the decline of synapsis development and glucose metabolic process, which is essential in neuron development. The GE is essential in the early development of nervous system.

Glycolysis is required to maintian the pluripotency of hESCs (Shyh-Chang and Daley, 2015). Previous studies have shown multiple pathways that could play important roles in promoting glycolysis in ESCs. For example, the stemness factor SALL4 can promote glycolysis by inducing the expression of HIF1a and GLUT1 (Kim et al., 2017). In addition to maintaining the genomic stability of ESCs (Lin et al., 2005; Xu, 2005), the p53-PUMA pathway suppresses the oxidative phosphorylation by limiting pyruvate uptake into the mitochondria (Kim et al., 2019). As the core transcription factors to maintain the pluripotency of hESCs, the knockdown of NANOG, OCT4 and SOX2 will lead to rapid differentiation and death of hESCs, making it difficult to study the roles of SON in regulating the expression of GLUT1 in pluripotent state (Avilion et al., 2003; Mitsui et al., 2003; Ivanova et al., 2006). In this context, previous studies have failed to provide conclusive data on the roles of SON on the regulation of GLUT1 expression. Our data indicated that the mutation of SON binding motif decreases the expression of GLUT1 by disrupting the interaction between the enhancer and promoter of GLUT1. Therefore, SON plays important roles in activating the enhancer of GLUT1 by directly binding to it.

Enhancer activity is often cell type-specific (Pennacchio et al., 2013). As expected, the epigenetic signatures of GE in iPSCs are similar to those in hESCs. The published ChIP-Seq data indicate that OCT4 binds to GE in iPSCs (Fig. 4A). Therefore, it can be speculated that SON activate the transcription of GLUT1 by binding to GE. While the epigenetic signatures of some human cancer cell lines such as HepG2 and HCT116 indicate that GE is active in these cell lines, the shape of the peak signal of H3K27ac is different from that of ES and IPSCs, suggesting that the transcription factors other than SON might be in involved in activating GE in human cancer cells. In addition to GE, the analysis of the epigenetic signatures of enhancers in these human cancer cells identifies multiple potential enhancer elements for GLUT1, suggesting that the expression of GLUT1 might be regulated by multiple enhancer elements in human cancer cell cells.

## MATERIALS AND METHODS

### Genome editing of hESC culture

H1 and H9 hESC lines were maintained on matrigel-coated plates in complete mTeSR™1 medium, and passaged using accutase or ReleSR. All reagents were obtained from STEMCELL Technologies. The CRISPR/Cas9 technology was used to edit the genome of hESCs as previously described (Rong et al., 2014). For the knockout of GE in hESCs, two expression cassettes encoding the sgRNA sequences (sgRNA1-GE: GGAAAAGGCTGGGAGGCCAG, sgRNA2-GE: GGCTGCTGTGATGCTCGAAT) flanking the deletion region were cloned into a plasmid that expresses a codon-optimized version of Cas9. For the mutation of the SON binding site within GE, the expression cassette encoding one sgRNA (sgRNA-SON: GGAACCTTTGTCATTCAAAC) targeting the SON motif was cloned into a plasmid that expresses a codon-optimized version of Cas9. To transfect the plasmid into the hESCs, hESCs were harvested using accutase and nucleofected in 4D-Nucleofector® Solution. The transfected hESCs were selected with puromycin and individual clones expanded and genotyped. The genotyping primers are following: 5′-AGGTCTCCCAAGTCTAGCGT-3′, 5′-TGATTACCGCAAAGCCCCAA-3′, 5′-CCCAAAACAGGGGATCCTGAA-3′.

### The analysis of ChIA-PET and ChIP-seq data

The ChIA-PET and ChIP-seq data were obtained from Gene Expression Omnibus (GEO) and Encyclopedia of DNA Elements (ENCODE). The accession were GSM1505699, GSM1505728, GSM1565766, GSM2534369, GSE57913, GSE44288, GSM1000126, GSE29611, GSE69643 and GSE69646 (Consortium, 2012; Whyte et al., 2013; Dowen et al., 2014; Pope et al., 2014; Yue et al., 2014; Tsankov et al., 2015; Ji et al., 2016). The interaction data derived from cohesin ChIA-PET analysis were displayed in BED12 format that showed the anchors and coordinates of the loop (Ji et al., 2016). The insulator loops were colored in red and the others in green. The ChIP-Seq data were displayed using the UCSC Genome Browser (http://genome.ucsc.edu/). All analyses of hESCs were performed using human (build hg19, GRCh37) RefSeq annotations downloaded from the UCSC genome browser.

### Analysis of gene expression profile

The gene expression profiles of hESCs after the knockdown of NANOG, OCT4 and SOX2 were downloaded from GEO (accession GSE34904) and analyzed by Qlucore Omics Explorer 3.3 (http://www.qlucore.se/) (Wang et al., 2012). *P*-value (two-tailed) was calculated with two-group comparisons.

### Chromosome conformation capture (3C) analysis

The 3C libraries for the cross-linked chromatin of hESCs were generated as described previously (Hagege et al., 2007; Hao et al., 2015; Deng and Blobel, 2017). The restriction enzyme *Eco*RI was chosen to digest the genome of hESCs. As the internal control, BAC vector (ctd-2542n5 from Invitrogen) covering the GE-GLUT1 gene locus was digested with *Eco*RI and religated. The frequency of interaction between the anchoring point and distal fragments was determined by TaqMan qPCR (StepOnePlus, Applied Biosystems, AB) and normalized to the BAC template. Normalization of 3C data from different samples was done by arbitrarily setting the non-specific interaction of the bait fragment with one of its nearest neighbor fragment to 1. Sequences of primers and probes are provided below.

**Table:** 

3C primers	Sequence
ge-ECORI-f-bait	CCTCGCCTCCCAAAGTACTG
ge-ECORI-f-probe	ATTACAGGCGTGAGCCACTGAGCCC
ge-ECORI-f-1	CCAGGTAAGTGATTGGTATGGAGTT
ge-ECORI-f-2	GAGTCCAAGGAAGCAAGAAATATT
ge-ECORI-f-m-1	CGCCAAAGAAGAAAACAATTACC
ge-ECORI-f-m-2	CGTCTCAACTGGATTATCAGATAGG
ge-ECORI-f-m-3	GGAGTGCCTAGGGTTTTCTATCC
ge-ECORI-f-p	GAGGGCTGTAAGGGAGAATCC
ge-ECORI-f-p-1	GCAACGATGTTGGAGTATTTGTC

### Quantitative RT-PCR

Real-time PCR was performed as previously described (Zhang et al., 2014). Briefly, total RNA was purified from hESCs with RNeasy Mini Kit (QIAGEN), and total RNA (1 µg) was reversely transcribed into cDNA and analyzed by qPCR. The primers for β-actin are 5′-GCCAACACAGTGCTGTCT-3′ (forward primer) and 5′-AGGAGCAATGATCTTGATCTT-3′ (reverse primer). The primers for GLUT1 are 5′-CTTTGTGGCCTTCTTTGAAGT-3′ (forward primer) and 5′-CCACACAGTTGCTCCACAT-3′ (reverse primer). The primers for NANOG are 5′-CATGAGTGTGGATCCAGCTTG-3′ (forward primer) and 5′-CCTGAATAAGCAGATCCATGG-3′ (reverse primer). The primers for OCT4 are 5′-AGTGAGAGGCAACCTGGAGA-3′ (forward primer) and 5′-ACACTCGGACCACATCCTTC-3′ (reverse primer). The primers for SOX2 are 5′-TGGACAGTTACGCGCACAT-3′ (forward primer) and 5′-CGAGTAGGACATGCTGTAGGT-3′ (reverse primer). The levels of GLUT1 mRNA were normalized to those of β-actin. Changes in mRNA expression were calculated according to the 2^−ΔΔCT^ method (CT, cycle threshold).

### Glycolysis analysis

Extracellular acidification rate (ECAR) was measured with the Seahorse XFe96 Analyzer (Seahorse Bioscience). H1 cells (2 × 10^4^/well) were seeded in Matrigel-coated 96-well XF Cell Culture Microplate and incubated overnight. The next day, cells were pre-incubated in XF assay media (supplemented with 2 mmol/L L-glutamine) for one hour prior to the assay. Glycolysis Stress Test was performed following manufacturer’s protocol. The obtained ECAR was normalized by fluorescence intensity of DAPI stained nuclei and analyzed using the XF Report Generator (Seahorse Bioscience).

### ChIP qPCR assay

Chromatin immunoprecipitation (ChIP) assays were performed using a SimpleChIP Enzymatic Chromatin IP kit (No. 9003; Cell Signaling Technologies). Briefly, hESCs were cross-linked with 1% formaldehyde at room temperature for 10 min. Chromatin was treated with micrococcal nuclease, sonicated, and immunoprecipitated with rabbit anti-acetyl-Histone H3 (Lys27) antibody (8173s; CST), rabbit anti-mono-methyl-Histone H3 (Lys4) antibody (5326s; CST), rabbit anti-Nanog (D73G4) antibody (5232s; CST), rabbit anti-Oct-4 (C30A3C1) antibody (5677s; CST), rabbit anti-Sox2 antibody (D6D9) (5024s; CST), and normal rabbit IgG (negative control) (2729; CST). After the reverse cross-linking and DNA purification, immunoprecipitated DNA was quantified by qPCR (5′-GGTTCTTTCTTCCACCGCGT-3′ and 5′-AGCAAGAATCCCAACCCCG-3′).

### Western blot

Western blotting analysis was performed as we previously described (Kim et al., 2015). Monoclonal antibodies used: anti-Glut1 (ab150299; Abcam) and anti-β-Actin (ab8227; Abcam). The intensity of the bands was quantified using Image Lab software.

### Homologous analysis

GE sequence from various species was compared using BLASTN 2.9.0 in NCBI. The algorithm of Fast Minimum Evolution was used to produce the tree from given distances between sequences of species (Desper and Gascuel, 2004).

### Statistics

GraphPad Prism 5 was used for statistical analysis. For comparisons between two groups of equal sample size, an unpaired two-tailed *t* test was performed. For comparisons of two groups of paired samples, paired two-tailed *t* test was performed. *P* < 0.05 was considered to be statistically significant.

### Teratoma formation

For teratoma fomation of hESCs in immunodeficient mice, 1.5 × 10^6^ hESCs and GE-deleted hESCs were harvested, washed twice with PBS, suspended in PBS with 30% Matrigel, and subcutaneously injected into region around the right (WT hESCs) and left (GE-KO hESCs) hind legs of immunodeficient mice. The teratomas were recovered 40 days after transplantation. Total RNA was purified from the teratomas with Trizol and processed for RNA-seq. All institutional and national guidelines for the care and use of laboratory animals were followed.

### RNA-seq and analysis

RNA purity was checked using the kaiaoK5500®Spectrophotometer (Kaiao, Beijing, China). RNA integrity and concentration were assessed using the RNA Nano 6000 Assay Kit of the Bioanalyzer 2100 system (Agilent Technologies, CA, USA). A total amount of 2 μg RNA per sample was used as input material for the RNA sample preparations. Sequencing libraries were generated using NEBNext® Ultra™ RNA Library Prep Kit for Illumina® (#E7530L, NEB, USA). Paired-end sequencing was completed on an Illumina HiSeq system. Clean data were renerated after removing adapters and low quality reads. The reference GRCh38 genomes and the annotation file were downloaded from ENSEMBL database (http://www.ensembl.org/index.html). Bowtie2 v2.2.3 was used for building the genome index, and Clean Data was then aligned to the reference genome using HISAT2 v2.1.0. Reads Count for each gene in each sample was counted by HTSeq v0.6.0, and FPKM (Fragments Per Kilobase Millon Mapped Reads) was then calculated to estimate the expression level of genes in each sample. The differentially expressed genes were analyzed with Qlucore Omics Explorer 3.3. Gene ontology (GO) biological process enrichment was analyzed by DAVID (https://david.ncifcrf.gov).

## Electronic supplementary material

Below is the link to the electronic supplementary material.

Supplementary material 1 (PDF 271 kb)
