# Supplementary material for: Core pluripotency factors promote glycolysis of human embryonic stem cells by activating GLUT1 enhancer
Source: Protein Cell. 2019 May 31;10(9):668–80. doi: 10.1007/s13238-019-0637-9 (PMC6711954; doi:10.1007/s13238-019-0637-9)
Supplement: Supplementary file 1 — Supplementary material 1 (PDF 271 kb) [file 13238_2019_637_MOESM1_ESM.pdf]

Supplemental Fig. 1. The activity of GE in H1 hESCs.

A. GE interacts with the promoter of the GLUT1 gene in H1 hESCs as confirmed by 3C assay. GE exhibited the enhancer-specific epigenetic markers in H1 hESCs. The GLUT1 promoter is shaded dark blue and the target restriction fragments shaded light blue. The GE-containing fragment is shaded pink. Relative cross-linking data for each restriction fragment was plotted. Data are represented as mean ± SD. N=3.

B. The primers to genotype the GE-KO H1 hESCs are indicated.

C. The PCR analysis confirmed the homozygous disruption of GE in GE-KO H1 hESC lines.

D. The deletion of GE in H1 hESCs reduced the mRNA levels of *GLUT1* gene. Data are represented as mean ± SD. N=3.

E. Sequencing analysis confirmed the deletion of 1361bp GE in GE-KO hESCs.

F. Knockdown of SOX2, OCT4, or NANOG alone did not affect the expression of the *GLUT1* mRNA. The heatmap of the gene expression after the knockdown of NANOG, OCT4 and SOX2 in hESCs was shown on the left, and the expression of specific genes on the right. The gene expression profiles of hESCs after the knockdown of NANOG, OCT4 and SOX2 were downloaded from GEO (accession GSE34904). Data are represented as mean ± SD. N=8.

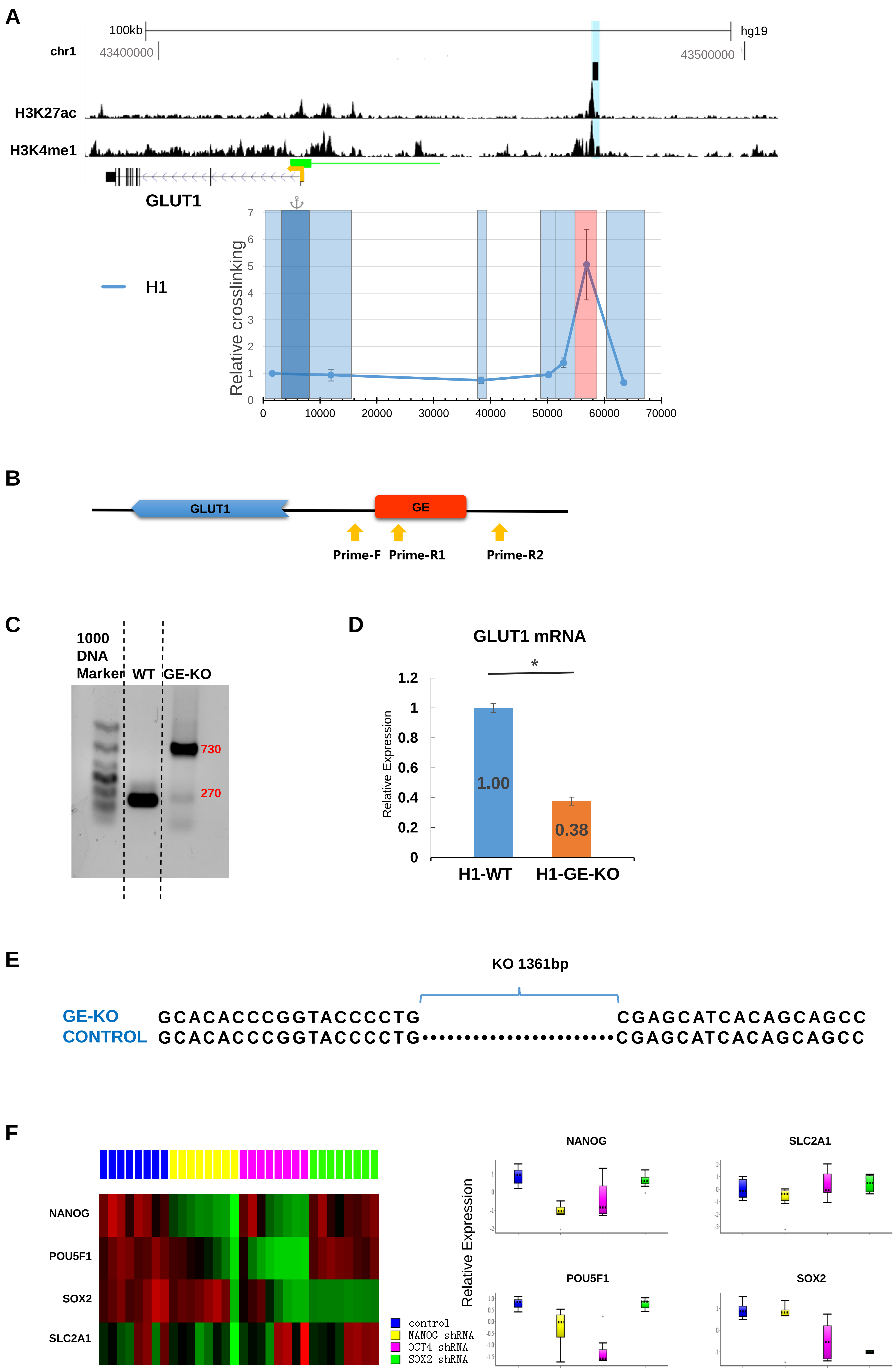

Supplemental Figure 1
